# Supplementary material for: Development of a computer-aided design software for the quantitative evaluation of aesthetic damage
Source: PLoS One. 2019 Dec 18;14(12):e0226322. doi: 10.1371/journal.pone.0226322 (PMC6919621; doi:10.1371/journal.pone.0226322)
Supplement: S1 Code site — 1. (ZIP) [file pone.0226322.s001.zip › [EESC_jr][Projeto]Relatorio_07_21/reports/templates_zip/gentelella-master/vendors/morris.js/examples/decimal-custom-hover.html]

# Decimal Data

```
var decimal_data = [];
for (var x = 0; x <= 360; x += 10) {
  decimal_data.push({
    x: x,
    y: 1.5 + 1.5 * Math.sin(Math.PI * x / 180).toFixed(4)
  });
}
window.m = Morris.Line({
  element: 'graph',
  data: decimal_data,
  xkey: 'x',
  ykeys: ['y'],
  labels: ['sin(x)'],
  parseTime: false,
  hoverCallback: function (index, options, default_content, row) {
    return default_content.replace("sin(x)", "1.5 + 1.5 sin(" + row.x + ")");
  },
  xLabelMargin: 10,
  integerYLabels: true
});
```
